# Patients’ preconceptions of acupuncture: a qualitative study exploring the decisions patients make when seeking acupuncture

**DOI:** 10.1186/1472-6882-13-102

**Published:** 2013-05-13

**Authors:** Felicity L Bishop, George T Lewith

**Affiliations:** 1 Centre for Applications of Health Psychology, Faculty of Social and Human Sciences, Building 44 Highfield Campus, University of Southampton, Southampton, SO17 1BJ, UK; 2 Primary Care and Population Sciences, Aldermoor Health Centre, University of Southampton, Aldermoor Close, Southampton, SO16 5ST, UK

**Keywords:** Placebo, Context, Acupuncture, Health care utilisation, Complementary medicine, Expectations, Health knowledge attitudes practice, Illness behaviour, Qualitative research, Patient preference, Treatment seeking

## Abstract

**Background:**

Like any other form of healthcare, acupuncture takes place in a particular context which can enhance or diminish treatment outcomes (i.e. can produce contextual effects). Patients’ expectations of acupuncture might be an important component of contextual effects, but we know relatively little about the origins and nature of patients’ expectations or wider preconceptions about acupuncture. Our aim was to identify the processes the underpin patients’ decisions to try acupuncture and thus begin to tease out the origins and nature of patients’ preconceptions.

**Methods:**

One-off semi-structured interviews were conducted with a purposive, varied sample of 35 adults who had tried acupuncture for various conditions. Interviews explored people’s experiences of acupuncture treatment and techniques from framework and inductive thematic analysis were used to relate the data to the research question.

**Results:**

We identified four distinct processes within participants’ accounts of deciding to try acupuncture: establishing a need for treatment, establishing a need for a new treatment, deciding to try acupuncture, and finding an acupuncturist. Family, friends and health care professionals played a role in these processes, providing support, advice, and increasing people’s general familiarity with acupuncture. When they came to their first acupuncture appointment, participants had hopes, concerns, and occasionally concrete expectations as to the nature of acupuncture treatment and its likely effects.

**Conclusions:**

Existing theories of how context influences health outcomes could be expanded to better reflect the psychological components identified here, such as hope, desire, optimism and open-mindedness. Future research on the context of acupuncture should consider these elements of the pre-treatment context in addition to more established components such as expectations. There appears to be a need for accessible (i.e. well-disseminated), credible, and individualised, patient-centred materials that can allay people’s concerns about the nature of acupuncture treatment and shape realistic hopes and expectations.

## Background

Like any other form of healthcare, acupuncture takes place in a particular context which can enhance or diminish treatment outcomes. According to one framework, this context comprises the patient’s characteristics, the acupuncturist’s characteristics, the patient-acupuncturist relationship, the treatment’s characteristics, and the setting [1,2]. Some researchers have gone further, suggesting that acupuncture is not just “any other form of healthcare” but rather has enhanced context effects which are sometimes referred to as large non-specific or placebo effects [3-5]. For example, Lewith and colleagues estimated that the “specific” effect of acupuncture (the effects of needle penetration and placement) contributes just 10% of its overall effect [6]. A recent individual patient data meta-analysis confirms that while acupuncture is clinically effective for chronic pain the difference in effectiveness between true acupuncture and placebo acupuncture is small compared to the difference between true acupuncture and non-acupuncture controls [7]. Again, this suggests contextual factors (shared across true and placebo acupuncture) make a large contribution to the overall effectiveness of acupuncture. A contextual factors framework is suited to understanding acupuncture as a complex intervention not least because it does not require us to make a problematic reductionist distinction between specific and non-specific effects [8-11]. Instead, this framework allows us to foreground the contextual milieu of treatment without denying that processes such as diagnosis are firmly embedded in and interact with that milieu.

In this paper, we focus on the “patients’ characteristics” component of context. Previous studies have identified some personal and psychological characteristics that might predict or mediate positive acupuncture outcomes. Across four large German trials of acupuncture for chronic pain the effects of acupuncture were enhanced for women, people living with others, previous positive experiences of acupuncture and previous negative experiences of other treatments [12]. Individual studies have failed to find significant relationships between personal characteristics and acupuncture effectiveness but these may have lacked power to detect small effects of single variables [13-15]. Turning to more psychological characteristics, patients’ expectations have received much attention [16-20]. In a classic trial comparing acupuncture and massage for low back pain, patients who expected better outcomes from acupuncture were more likely to experience better outcomes from acupuncture (compared to massage) [17]. Across the four large German trials, higher expectations predicted better outcomes [18] but other studies do not find a significant relationship between expectations and outcomes [19]. Other psychological factors such as patients’ illness perceptions and self-efficacy might mediate acupuncture outcomes, but few studies have tested these hypotheses [21].

In summary, quantitative evidence suggests that patients’ expectations of acupuncture might be an important component of contextual effects. This is consistent with the findings from a major review that expectations related to medical procedures, self-management, and treatments are related to health outcomes and should be enhanced in clinical practice [22]. However, we know relatively little about the origins and nature of patients’ expectations about acupuncture. A better understanding of the context surrounding the start of treatment could indicate opportunities to enhance the contextual effects of acupuncture. We therefore conducted a qualitative analysis to identify the processes that underpin patients’ decisions to try acupuncture and the origins and nature of patients’ expectations. We chose a qualitative approach to help us to access the content and nature of participants’ views without being constrained by existing constructs such as expectancies [23]. This paper describes the decision-making processes of patients choosing to try acupuncture, examines the nature of their expectations about acupuncture, and draws out implications for understanding context effects.

## Methods

Ethics approval was obtained from the University of Southampton School of Psychology ethics committee (ST/03/92).

### Data collection and participants

One-off semi-structured interviews were used to explore people’s experiences of acupuncture treatment. Semi-structured interviews were conducted in person by the first author in a location of the participant’s choosing (in most cases their home, occasionally their work place) during 2007. The topic guide was designed to explore the context of acupuncture from patients’ perspectives, construed broadly as beginning from before the first treatment and extending throughout patients’ experiences of acupuncture (Table 1).

**Table 1:** Summary of interview topic guide

**Description**	**Question(s)**
Main question	I’m really interested in finding out why you chose to have acupuncture and what it has been like now that you have tried it. Please could you tell me all about it?
Supplementary questions – Only used if needed to encourage the participant to fully describe their acupuncture experience(s)	How did you come to try acupuncture?
	What did you think having acupuncture would be like?
	Could you tell me all about the last acupuncture treatment that you had?
	Could you tell me about the person who gives you your acupuncture?
	Could you tell me about the place where you have your acupuncture?
	How do you feel about your experience of acupuncture so far?
	How do you think acupuncture works?
Final question	Is there anything else that you would like to tell me about your experiences or understanding of acupuncture?

We tried to recruit participants who would give us varied accounts of acupuncture, in order to map a range of contextual components and, for this analysis, a range of pathways into acupuncture. We recruited patients from diverse settings and with diverse experiences of acupuncture following a purposeful strategy of sampling for maximum-variation [24]. Maximum-variation sampling was used partly to identify any extreme experiences but primarily to identify common features shared across diverse participants, which thus form a central, essential, core of the decision-making processes we were investigating. Twenty six participants were recruited from 7 acupuncturists working in private practice in the South of England; 9 were recruited through an advertisement placed on a University website asking for people who had experienced “disappointing, unsuccessful or negative experiences of acupuncture”. These two recruitment processes occurred in parallel as we anticipated that recruiting participants through clinical settings alone might mean we only heard from people who had positive experiences of treatment. This expectation was borne out as only a few participants whom we recruited through clinical settings described earlier, disappointing experiences of acupuncture with other acupuncturists. Recruiting from the University community also enabled us to achieve greater diversity in terms of the settings in which our participants had experienced acupuncture, including, for example, one participant who had experienced acupuncture on a cruise ship.

Six men and 29 women aged between 26 and 86 years (median 53 years) volunteered and gave informed consent to take part in an audio-recorded interview. They had experienced acupuncture treatment for various conditions, including: hay fever, rheumatoid arthritis, anxiety, fertility/IVF, IBS, digestive problems, sinusitis, shoulder/neck pain, tendonitis, smoking, low energy levels, menopause, menstrual problems, fibromyalgia, wheat intolerance, stress, osteoarthritis, headache, good health maintenance, lost voice, flying phobia, joint pain, back pain, tight muscle in thigh, and post-viral syndrome. Five participants had experienced acupuncture in public-sector clinics. The extent of participants’ experiences of acupuncture ranged from 1 treatment session to 25 years’ of experience. The interviews lasted between 24 minutes and 2 hours (median duration was 53 minutes).

Data analysis began immediately after the first interview was conducted and subsequent interviews and analysis proceeded iteratively. We stopped recruiting when categories and themes were well-developed in relation to our research questions, new participants’ accounts were very similar to earlier participants’ accounts and it was felt that additional insights were unlikely to be generated without considerably expanding the sampling frame (e.g. to a distant geographical area) [25,26].

### Data analysis

**Figure 1:**
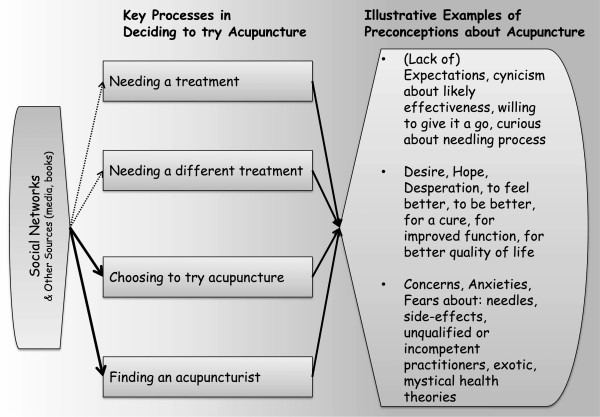
**Thematic Map.** Showing the decision-making processes that lead patients to try acupuncture and the nature of the resulting preconceptions about treatment process and outcomes.

All interviews were audio-recorded with each participant’s consent and then transcribed verbatim; identifying details were disguised as soon as possible after conducting each interview. We used techniques from framework analysis and inductive thematic analysis to code and interpret the data [27-29]. We began by developing a thematic framework to organise patients’ talk into the five domains of contextual features: patients’ characteristics, acupuncture, their acupuncturist, their relationship with their acupuncturist, and the physical and institutional setting of acupuncture. Across these domains, we also distinguished between talk about experiences prior to acupuncture and those that occurred during and after the first acupuncture consultation, i.e. we applied a timeframe. We applied this framework to the data as each interview was conducted and transcribed, i.e. we indexed segments of patients’ talk according to domain and timeframe. We then created a detailed summary chart which consisted of a series of columns (one for each domain in each timeframe) and rows (one per participant). Each cell contained a summary, relevant verbatim extracts and references to the location of relevant material in the original interview. Patterns in the data were explored inductively to identify components and processes that constitute the context of acupuncture as experienced by patients. This exploration continued iteratively alongside data collection as the indexing and charting was carried out for each interview in turn. The findings below are based on a focused review of the chart contents related to experiences leading up to the first consultation. A thematic map (Figure 1) and narrative description of the themes was produced and analysed in relation to existing literature and theory. At this point, it became apparent that the data could be interpreted with reference to existing theory, namely, Leventhal’s common-sense model of illness perception [30,31]. We therefore reviewed this theory and used it to help us name and understand some of the patterns we had identified in the data. Quotes presented below have been selected for typicality and eloquence in illustrating a particular analytic point; speakers are identified by pseudonym.

### The role of the researchers

We took a critical realist approach to this work. Our ontological assumption is that while we cannot objectively observe participants’ experiences by attending to their accounts, we can treat their accounts as a representation of their experiences. Epistemologically, we assume that the interviewer has an unavoidable role in shaping the interviewee’s accounts (for example merely asking a question invites the interviewee to produce an account of a particular experience) and that the analyst similarly brings existing theories and knowledge to bear when interpreting interviewees’ accounts. Methodologically, we assume that by adopting rigorous analytic procedures in a reflexive way we can lessen inappropriate influences of the researchers in the production of what is necessarily culturally-bound knowledge. By presenting the topic guide and clearly indicating where existing theories and knowledge have informed the analysis we hope to render these influences transparent and thus accessible for review by the reader.

## Results

As shown in the thematic map (Figure 1) we identified four processes that took participants from not using acupuncture to having booked a first appointment: establishing a need for treatment, establishing a need for a new treatment, deciding to try acupuncture, and finding an acupuncturist. Each of these processes was social and involved family, friends, and/or health care professionals and together they contributed to patients’ expectations, desires, and hopes about the process and outcomes of acupuncture.

### Establishing a need for treatment

Participants talked about an embodied need for treatment and their descriptions strongly resonated with established dimensions of illness perceptions. Leventhal’s common-sense model of illness perception suggests that people’s illness cognitions are structured along five core dimensions: illness identity, timeline, consequences, causes, and controllability; symptoms can also trigger *emotional* representations which are somewhat independent of the cognitive dimensions [30,31]. We identified the first four dimensions in participants’ talk about a need for treatment, while the fifth (controllability) was present in talk about needing a different treatment (see below).

The *illness identity*[30,31] of participants’ need for treatment could incorporate a variety of symptoms and difficulties which sometimes had a specific precipitating event (i.e. *causes*[30,31]) or duration (i.e. *timeline*[30,31]). Andrew had a specific localised physical symptom that he sought acupuncture for: “like my stomach would blow up and just feel really uncomfortable. That is what I went to him for initially.” Katherine’s need for treatment was more widespread encompassing physical and psychological issues “back ache, and tired and stressed and a bit low actually.” The *illness identity*[30,31] of Jackie’s need for treatment was difficult to name and she described struggling to obtain a diagnosis for long-standing symptoms (a chronic *timeline*) which she subsequently labelled post-viral syndrome: “basically I was ill over a year ago now with a virus and nobody could come up with any solution to my problem.” Linda described coming to an understanding of the *identity* of her illness (thinking it was bunions then arthritis), its *timeline* (chronic) and its perceived *causes* (hereditary).

“I’ve got arthritis very badly and I’ve had it for many years. My husband died about 35 years ago I think now, bless him, and it was about 18 months after that I started getting arthritis. And I went to the hospital thinking I had bad toes, I thought I had bunions and they laughed at me, they said, no it’s definitely arthritis because it’s in the family, it was in my….my mother had it badly.” (Linda)

Other participants identified psychological issues, such as Timothy who had fear of flying, and Annie who was the primary caregiver for her husband who had Parkinson’s disease.

“I think really you can pin it down to my husband’s trouble why I first went. Maybe I would have put up with stress and that sort of thing, but it was really because of him that I went.” (Annie)

Participants described their condition as having at times severe and in some cases wide-ranging *consequences*[30,31] for them that impacted their wellbeing and quality of life and which drove them to seek treatment. Nelly felt “suicidal by the time I started going” which illustrates the theoretical assertion that symptoms are represented emotionally (as well as cognitively) and that these emotional representations can trigger help-seeking [30,31]. Less emotively, Carl described how his thumb pain needed to be treated because it was stopping him learning to drive. Jessica described the perceived *consequences* of her illness when she talked about how her hormonal arthritis impacted her family life and daily activities:

“It affected my hands and my feet and my ankles and wrists most of the time, so I couldn’t do anything. This was just at the time that I had a 5 year old child who’d just started school.” (Jessica)

In summary, participants started acupuncture when they were experiencing uncomfortable, painful, inconvenient, impairing, or distressing condition(s) or symptom(s). They started treatment with a strong desire or motivation to get better whether that was seen in terms of recovery, relief, and/or improved control.

### Establishing a need for a different treatment

When talking about needing a different treatment, participants were describing their perceptions of their own or their previous treatment’s limited ability to control*,* manage, or cure, their condition. This talk therefore mapped onto the fifth theoretical dimension of illness perception, perceived *controllability*[30,31]. Participants discussed how whatever they had done before acupuncture was somehow unsatisfactory; hence they needed to try a different or new treatment to improve the *controllability*[30,31] of their condition. Overlapping with previously documented “push” factors thought to encourage people to use complementary therapies [32], we identified three situations in which participants described needing a different treatment.

First, when they had tried or been offered treatments which offered relief but also inspired concerns about side-effects, potential addiction or increased tolerance. For example, Belinda described her experience of side-effects from anti-histamines which eventually led her to seek an alternative treatment: “I used to have to take a lot of anti-histamines and really just drug myself up and just keep stepping up the dose as the season went on, until I was just a zombie.” Ken found the prospect of cortisone injections unacceptable:

“I went to see the doctor and he said, ‘you need to rest it’. I said, ‘I have rested it’. He said, ‘alright, we’ll give you cortisone injections’. And I said ‘no you won’t. I don’t believe in taking chemicals unless you really have to’.” (Ken)

Second, when previous treatment(s) had neither controlled nor cured a condition. Jessica had tried conventional treatments for her arthritis, but “a very clever senior doctor had tried his very best with everything available to him and hadn’t, you know, hadn’t managed anything.” It was not only previous conventional treatments that had proven unsuccessful for our participants. Timothy had tried a long list of treatments to cure his fear of flying, including homeopathy, reiki, tapping, and a British Airways programme “all of which have been unsuccessful so I’d really sort of run out of options”.

Third, when they had been told there was nothing conventional medicine could offer. Katherine had not tried conventional medicines for her arthritic pain because she had been to her GP who had “said she couldn’t really do anything about the pain in my fingers and I thought well, I’m not really that happy about that because I’m only in my 40s and I’m not… I don’t want to be feeling like this.” Alison felt she had been dismissed when her GP told her to rest her voice and offered no treatment, which left her needing to seek an alternative: “I think I was pretty much at my wit’s end because having been told ‘oh just go and rest your voice and it will come back’ and I knew I was more seriously ill than that.”

In searching for disconfirming cases we identified two participants, Betty and Grace, who had not tried anything else for a particular condition before trying acupuncture: they did not talk about needing a different treatment but focused entirely on deciding to try acupuncture. This might represent a newer pattern of use, in which acupuncture is used as a first line treatment possibly by patients who already have some familiarity with it. Both Betty and Grace had used other complementary therapies before acupuncture and both tried acupuncture at clinics they were already attending for other treatments.

In summary, participants typically but not always came to acupuncture having tried other treatments. This often extensive history of treatment-seeking gave some participants a desperate determination to find an effective treatment, while others appeared somewhat resigned to working though a diminishing number of treatment options.

### Deciding to try acupuncture

Having established a need for a different treatment, participants talked about making a specific decision to try acupuncture. Some participants saw acupuncture specifically as an attractive treatment that could offer them something that previous treatments had not provided. For some participants this was based on anecdotal evidence and success stories from friends and acquaintances, consistent with the role of lay referral networks identified in studies of CAM use in cancer [33,34]. A specific attraction to acupuncture resonates with the “pull” factors thought to encourage people to use complementary therapies [32]. It is also consistent with the suggestion from the common-sense model that people select treatments to try based on the extent to which they perceive that particular treatment as likely to help the condition at-hand [30,31]. However, other participants saw acupuncture as just another option, much like many other potential treatments, and these participants placed little emphasis on any attractive characteristics of acupuncture specifically. In other words, patients varied in the extent to which they described a specific pull towards acupuncture, rather than a general push away from other treatments that they had already tried.

Participants who described feeling specifically attracted to acupuncture described a perceived fit between acupuncture and themselves and/or their condition. Such perceptions were based on ideas from friends, family members, colleagues, books, and other mass media. Betty had read a book on acupuncture and intuitively felt that it was appropriate for her condition: “Because I had a very very tight and uncomfortable muscle in my thigh and I just felt that maybe it needed something to stick needles in it to sort of wake it up.” Jessica was also pulled specifically towards acupuncture, but she focused on its non-pharmacological nature:

“[I was] keen to try something that wasn’t another drug. You know by that stage I just felt my body was being so bombarded by so many different drugs you know…I don’t think you could find many drugs which have as few side-effects as acupuncture has and that was very attractive.” (Jessica)

Participants who were not strongly pulled towards acupuncture did not clearly differentiate it from other potential treatment options. For example Timothy chose acupuncture “purely by coincidence” after coming across a traditional Chinese medicine clinic when out shopping and realizing that acupuncture might help his fear of flying. He did not talk about feeling attracted to acupuncture specifically, but decided to try it because he had already exhausted many other potential treatments. Other participants demonstrated a sense of ambivalence towards acupuncture as nothing special, just another treatment that was worth trying: “I was in pain, I needed to do something” (Ken). This ambivalence is more difficult to interpret in terms of the common-sense model as it suggests that any treatment might be considered viable regardless of whether it is perceived to be a good fit for the patient’s illness. Indeed, it suggests that choosing a treatment might have less to do with the perceived characteristics of the particular treatment and instead be motivated by a desire to avoid a negative outcome (i.e. the continuing symptoms that are anticipated if one does not seek a new treatment). This is broadly consistent with expectancy-value models (e.g. Theory of Planned Behaviour [35]) in which people are hypothesised to compare perceived outcomes of different actions in terms of likelihood and subjective value. Expectancy-value models would suggest that patients will seek acupuncture - even if they do not expect it to benefit them - when they believe that there will definitely be greater negative consequences of the alternative, i.e. *not* seeking acupuncture.

In summary, participants who were pulled towards acupuncture initiated treatment believing that it would be right for them: they believed it was a good match to their health problem and so should be effective, or that at least it was unlikely to result in the negative effects they had experienced with previous treatments. Participants who were not pulled towards acupuncture typically expressed more neutral beliefs about its possible effects, coming across as either open-minded or ambivalent.

### Finding an acupuncturist

We have described the process of finding an acupuncturist in detail elsewhere as it inspired a more focused research question and follow-up quantitative study [36]. In brief, participants were concerned to find an acupuncturist who would be a competent and personable clinician whom they would be able to trust to deliver an invasive acupuncture treatment and would feel at ease talking with. For most participants finding an acupuncturist was a fundamentally social process, and involved consulting family members, friends, colleagues, and/or directories in an attempt to ascertain whether an acupuncturist would be competent and trustworthy. A similar emphasis on peer referrals and recommendations was reported in a Canadian study of users of complementary medicine [37]. For this analysis, it is important to note that even after consulting their social networks some of our participants had residual doubts or concerns about their chosen acupuncturist’s competence and interpersonal style in the lead up to and even during their first appointment: “to be honest… I think I probably did lie on that table the 1st day and think oh I hope she’d passed all her exams.” (Ella.)

### Consequences of treatment-seeking processes

The processes of treatment-seeking described above resulted in diverse preconceptions about acupuncture which participants took with them as they first consulted their acupuncturist. When describing how they felt before their first acupuncture consultation, participants focused on anticipating the process of having treatment and its likely outcomes or effects.

### Anticipating the processes of treatment

Occasionally, participants anticipated having the acupuncturist perform tongue or pulse diagnosis and having a pleasant treatment setting. Much more typically, participants emphasised the needles when describing what they had anticipated acupuncture to be like. They expected acupuncture to involve needling and for some participants the thought of being needled caused some concern:

“I thought it would be bizarre and I thought it would be just somebody sticking pins all over me. I thought it would be very painful and I thought just the experience of laying, sitting, with the needles hanging out would be extremely uncomfortable.” (Timothy)

Other concerns about the process of treatment included concerns about whether the acupuncturist would be suitably skilful and concerns about the Eastern (and/or perceived mysterious) nature of acupuncture. For example, Shelly saw acupuncture as mysterious and was wary of trying it but knowing that her husband had benefitted from it encouraged her to overcome these concerns.

“You can sort of perceive it as being very kind of weird and sort of Eastern mysticism, and I don’t want to get involved with something that’s a bit strange. But [my husband] tried it so I thought oh well it can’t be that bad then.” (Shelly)

A few participants talked about having very little in the way of concrete expectations about the process of having acupuncture, which mirrored the neutrality seen in some decisions to try acupuncture. For example, Andrew said that “when you start off doing it you don’t know what it’s going to be like” while Annie said that “I really didn’t know, I went in with a very open mind.”

### Anticipating treatment outcomes

Participants typically described hoping for and wanting a range of treatment outcomes; talk about *expected* outcomes was rare. Some participants used vague language to describe how they wanted “help” with a condition but said they did not know enough about acupuncture before trying it to have formulated more precise ideas about possible outcomes. Participants were “willing to give it a go”, thought it was “worth a try” and were curious to “see what would happen”.

Some participants, particularly those who described having close friends or family members who had used acupuncture and were thus more familiar with it, described anticipating more specific effects. Typically, they described hoping and wanting acupuncture to reduce their need for treatment by alleviating symptoms and their associated consequences. The affective nature of this talk resonates with participants’ emotive accounts (described above) of the consequences of their health condition and their unsatisfactory experiences of other treatments.

“I hoped it would relieve the pain. I didn’t have any expectations of it improving the mobility but I wanted to get rid of the pain and if possible the swelling, to enable me to do what I wanted to do with my hands and feet.” (Kate).

Some participants expressed a strong (affective) desire for acupuncture to relieve their symptoms but held equally strong negative (cognitive) expectations about the likely outcome: “I was cynical, I didn’t think it would work at all, I was just desperate to get rid of the pain.” (Ken). Fay had unusually specific outcome expectations before starting acupuncture:

“I thought I’d stop aching. I really did. Because everybody had said to me oh it’s pain reduction, brilliant, you know, you’ve got to try it, and all the rest of it. I did expect after the first session I would walk out and you know my joints wouldn’t be as painful.” (Fay).

## Discussion

We identified four distinct processes within participants’ accounts of deciding to try acupuncture: establishing a need for treatment, establishing a need for a new treatment, deciding to try acupuncture, and finding an acupuncturist. Consistent with previous studies of complementary medicine use [33,34,37], family, friends and health care professionals played a role in these processes, providing support, advice, and increasing people’s general familiarity with acupuncture. Success stories, anecdotes and insights gleaned from friends and other social contacts encouraged some participants to try acupuncture and were valued sources when participants were trying to find an acupuncturist who would be competent and personable. Participants also consulted and were influenced by other sources such as books and mass media. When they came to their first acupuncture appointment, participants had hopes, desires, concerns, and occasionally concrete expectations as to the nature of acupuncture treatment and its likely effects.

We have illustrated how decision-making processes that lead to the initiation of acupuncture treatment help to shape and have implications for the nature and content of patients’ expectations of acupuncture, an important component of treatment context. The first consultation can be viewed as the culmination of a socially-informed decision-making process that contributes to patients holding a range of cognitive and affective expectations, hopes, desires, and concerns about both the process and possible outcomes of treatment. Elements of this context have been identified previously. For example, women in a trial of acupuncture for menopausal symptoms associated with tamoxifen hoped for particular outcomes as well as (or instead of) expecting them [38] as did women in a trial of acupuncture for polycystic ovary syndrome [39]. Patients receiving acupuncture as part of treatment for substance dependence were apprehensive about initiating an unfamiliar treatment [40] while adolescents in a trial of acupuncture for chronic pain were anxious about needling and thought it would be uncomfortable [41].

Existing theories of how context influences health outcomes could be expanded to better reflect the psychological components identified here. For example, cognitive expectations feature prominently in both theories of placebo effects [42,43] and acupuncture research [16-19]; our findings support suggestions that we should also be investigating the potential moderating and mediating roles of desperation, hope, [44] optimism [45], and desire [46]. Further conceptual work might be useful to understand the relationships between these constructs in healthcare settings, given that some of our participants seemed to have positive hopes and desires but negative expectations. Learning mechanisms, in particular conditioning, also feature heavily in theories of placebo effects [47]. Given our participants’ often extensive and disappointing previous experiences of treatment it would be useful to test whether conditioning is a plausible mechanism for contextual effects in real-world acupuncture treatments; perhaps the differences between acupuncture and other treatments are such that previously unsuccessful attempts at pain relief through other modalities are not generalised to acupuncture as a new stimulus. The ambivalence and open-mindedness displayed by some of our participants suggests that both state (i.e. context-dependent) and trait (i.e. enduring) versions of constructs such as openness to experience feature in the psychological context of acupuncture and might influence its effects [48].

In interpreting our data we drew on previous work about how people perceive illness [30,31] and the pull-push framework for conceptualising factors that attract patients to complementary therapies and repel them from conventional medicine [32]. Our findings are broadly consistent with the illness perception framework, suggesting that acupuncture patients do conceptualise their health problems (which were considerably varied) along established dimensions. In relation to the push-pull framework, our findings suggest that pull factors are more salient for some acupuncture patients (those who are specifically attracted to acupuncture) but push factors are more salient for those who see acupuncture as just one of a diminishing number of possible alternatives to manage a chronic problem. That two of our participants described choosing acupuncture without first describing a more general need for a new treatment is consistent with the suggestion from survey work that in recent years pull factors have become more important determinants of complementary therapy use [49].

Our analysis is limited by our reliance on one-off interviews with participants, which were conducted some time ago (2007). Also, while the majority of participants provided detailed accounts of initiating acupuncture a few could recall very little about their original decisions to try acupuncture as these decisions were made some years previously. Interviewing patients who have booked but not yet attended for their first consultation would enable us to test whether we have missed any important pre-treatment contextual factors. However, in our experience this is a difficult population to access for qualitative interviews as patients who are early in treatment can be reluctant to talk about their experiences. An open-ended written questionnaire item might better facilitate such a study [50].

## Conclusions

Future research on the context of acupuncture should consider the elements of the pre-treatment context identified here, in addition to more established components such as expectations. It would also be interesting to explore how acupuncturists orient to patients’ preconceptions of acupuncture and whether they inform the individualisation of treatment [51,52]. Of clinical relevance, we have identified an apparent need for accessible (i.e. well-disseminated) and credible patient-centred materials concerning the nature and likely outcomes of acupuncture. While credible printed materials are already available [53], future developments could take advantage of the opportunities for interactive and tailored materials offered by popular web-based delivery mechanisms such as social media and smart phones. Participants had concerns about needling and some had not formed concrete expectations about the process or outcomes of acupuncture. High quality, tailored, psychologically-informed patient education could help address concerns and start to manage patients’ expectations even before the first consultation. Doing so might be one way to enhance the psychological context of acupuncture and associated patients’ outcomes [22].

## Competing interests

The authors declare that they have no competing interests.

## Authors’ contributions

FLB conceived of the study, led its design, conducted the interviews and data analysis and drafted the manuscript for publication. GTL contributed to study design and data interpretation and helped to draft the manuscript. Both authors have given final approval of the version to be published.

## Authors’ information

FLB is lecturer in health psychology at the University of Southampton; she has a programme of research that encompasses utilisation of complementary and alternative medicines and context effects. GTL is professor of health research at the University of Southampton and leader of the Integrated Medicine research group; he has a long-standing research interest in acupuncture and has used it in his own clinical practice.

## Pre-publication history

The pre-publication history for this paper can be accessed here:

http://www.biomedcentral.com/1472-6882/13/102/prepub
